# Multifaceted Roles of Nerve Growth Factor: A Comprehensive Review with a Special Insight into Pediatric Perspectives

**DOI:** 10.3390/biology13070546

**Published:** 2024-07-19

**Authors:** Lavinia Capossela, Antonio Gatto, Serena Ferretti, Lorenzo Di Sarno, Benedetta Graglia, Miriam Massese, Marzia Soligo, Antonio Chiaretti

**Affiliations:** 1 Institute of Pediatrics, Fondazione Policlinico A. Gemelli IRCCS-Università Cattolica Sacro Cuore, 00168 Rome, Italy; serena.ferretti01@icatt.it (S.F.); lorenzo.disarno01@icatt.it (L.D.S.); benedetta.graglia01@icatt.it (B.G.); antonio.chiaretti@policlinicogemelli.it (A.C.); 2 Institute of Pediatrics, Fondazione Policlinico Universitario A. Gemelli IRCCS, 00168 Rome, Italy; antonio.gatto@policlinicogemelli.it (A.G.); miriam.massese@policlinicogemelli.it (M.M.); 3 Istituto di Farmacologia Traslazionale, Consiglio Nazionale delle Ricerche (CNR), 00133 Rome, Italy; marzia.soligo@ift.cnr.it

**Keywords:** nerve growth factor, neurotrophin, pediatric

## Abstract

**Simple Summary:**

Nerve growth factor (NGF) is a crucial protein for the growth and survival of neurons involved in various neuroprotective and regenerative processes. In neurological disorders, NGF plays a key role in maintaining neuronal health and function. In the context of traumatic brain injury (TBI), NGF levels increase in response to the damage, facilitating neuronal regeneration and reducing the extent of brain injury. These effects make NGF a promising candidate for therapies targeting both neurological diseases and traumatic brain injuries, offering hope for improving neurological function in affected patients.

**Abstract:**

Nerve growth factor (NGF) is a neurotrophic peptide largely revealed for its ability to regulate the growth and survival of peripheral sensory, sympathetic, and central cholinergic neurons. The pro-survival and regenerative properties of neurotrophic factors propose a therapeutic potential in a wide range of brain diseases, and NGF, in particular, has appeared as an encouraging potential treatment. In this review, a summary of clinical studies regarding NGF and its therapeutic effects published to date, with a specific interest in the pediatric context, will be attempted. NGF has been studied in neurological disorders such as hypoxic–ischemic encephalopathy, traumatic brain injury, neurobehavioral and neurodevelopmental diseases, congenital malformations, cerebral infections, and in oncological and ocular diseases. The potential of NGF to support neuronal survival, repair, and plasticity in these contexts is highlighted. Emerging therapeutic strategies for NGF delivery, including intranasal administration as well as advanced nanotechnology-based methods, are discussed. These techniques aim to enhance NGF bioavailability and target specificity, optimizing therapeutic outcomes while minimizing systemic side effects. By synthesizing current research, this review underscores the promise and challenges of NGF-based therapies in pediatric neurology, advocating for continued innovation in delivery methods to fully harness NGF’s therapeutic potential.

## 1. Introduction

The intricate network of neurons within the human brain orchestrates an array of cognitive, sensory, and motor functions, rendering it one of the most complex organs in the human body. Amidst this complexity, the role of neurotrophic factors, particularly nerve growth factor (NGF), emerges as a pivotal regulator of neuronal survival, development, and function.

The neurotrophins (NTs) are a family of trophic factors that play a major role in controlling crucial traits of development, survival, and the function of neurons in both the central and the peripheral nervous systems [1].

Nerve growth factor (NGF) is the first discovered member of the neurotrophin family. It was revealed and analyzed by Rita Levi-Montalcini, Viktor Hamburger, and Stanley Cohen in the early 1950s [2].

From a biochemical point of view, NTs are synthesized as pro-NTs that successively assume proteolytic cleavage and post-translational modifications [3]. These premature molecules essentially play an active role in being able to balance or adjust the function of the final forms. The complete form of NTs binds to high-affinity tropomyosin-related kinase (Trk) A, B, or C receptors or the low-affinity p75 pan-neurotrophin receptor (p75NTR). The TrkA receptor demonstrates the highest affinity for NGF [4,5]. The major cytosolic/endosomal pathways stimulated by the TrkA are Ras-mitogen-activated protein kinase (MAPK), extracellular signal-regulated kinase (ERK), phosphatidylinositol 3-kinase (PI3K)-Akt, and Phospholipase C (PLC)-γ [1]. The binding of NGF to p75NTR triggers additional signaling pathways that, in the absence of co-expressed TrkA, may lead to the apoptosis of the cell [6]. NGF formed by hippocampal and cortical neurons is known to bind TrkA and p75NTR and create a trimeric complex with NGF, leading to neuronal survival pathways [7,8,9,10].

NGF is essential for the development and phenotypic maintenance of neurons in the peripheral nervous system (PNS) and for the integrity of cholinergic neurons in the central nervous system (CNS) [11,12,13].

In the central nervous system (CNS), the highest amount of NGF is produced in the cortex, the hippocampus, and the pituitary gland, although important amounts of this neurotrophin are also produced in other areas, including the basal ganglia, thalamus, spinal cord and in the retina [14]. In the CNS, NGF also controls phenotypic features in noradrenergic nuclei of the hypothalamus and brainstem, contributing to the central regulation of autonomic response and the modulation of stress axis activity [15,16].

Its impact ranges beyond neurodevelopmental processes, embracing critical roles in neuroplasticity, synaptic transmission, and neuronal maintenance throughout life [1]. While historically studied in the context of neurodevelopment and neurodegeneration in adults, emerging research has highlighted the significance of NGF across a broad spectrum of pediatric brain disorders, traumatic brain injury (TBI), ophthalmology, and oncology.

In pediatric neurology, NGF is progressively recognized as a key player in the pathophysiology of various brain disorders affecting children, such as autism spectrum disorders (ASD), attention deficit hyperactivity disorder (ADHD), and pediatric epilepsy [17]. Dysregulation of NGF signaling pathways has been implicated in the abnormal neurodevelopmental trajectories detected in these conditions, suggesting potential insights into novel therapeutic strategies targeting NGF modulation [18].

Furthermore, traumatic brain injury (TBI) represents a significant cause of morbidity and mortality in pediatric populations, with long-term consequences ranging from cognitive impairments to behavioral disorders. In the aftermath of TBI, NGF orchestrates neuroprotective mechanisms, promotes neuronal survival, and facilitates neural repair processes, thus serving as a potential therapeutic target for mitigating the adverse sequelae of pediatric brain trauma [19].

In ophthalmology, NGF plays a crucial role in the development, maintenance, and function of sensory neurons innervating the eye. Perturbations in NGF signaling have been involved in various pediatric ophthalmic disorders, including congenital corneal anesthesia, neurotrophic keratitis, and congenital glaucoma [20]. Understanding the complexities of NGF-mediated neurotrophic support in pediatric ocular health holds promise for the development of innovative therapeutic approaches aimed at preserving vision and mitigating ocular complications.

Moreover, the sophisticated relationship between NGF and cancer biology has profound implications in pediatric oncology. While NGF exerts trophic effects on normal cells, dysregulated NGF signaling pathways have been implicated in the pathogenesis and progression of pediatric malignancies, including neuroblastoma and retinoblastoma [21,22,23].

Targeting NGF signaling pathways presents an encouraging opportunity for therapeutic intervention in pediatric oncology, with the potential to inhibit tumor growth and improve treatment outcomes.

**Figure 1:**
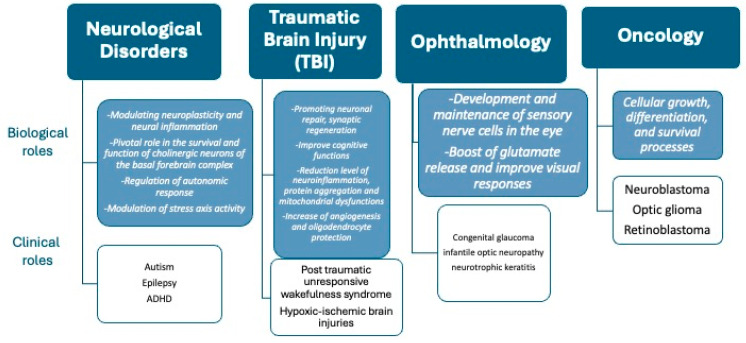
Biological and clinical roles of NGF in a pediatric perspective.

This comprehensive review aims to synthesize current knowledge regarding the multifaceted roles of NGF brain disorders, traumatic brain injury, ophthalmology, and oncology, focused on the pediatric age (Figure 1). By revealing the intricate interaction between NGF signaling pathways and pathological processes in pediatric populations, we try to overlay the way for the development of targeted therapeutic strategies aimed at improving the neurological, ophthalmic, and oncological outcomes of children worldwide.

## 2. Methods and Results

We conducted the literature research to find nerve growth factors-related studies and reports published between 1 January 1978 and 1 January 2024; the following electronic databases were systematically searched: PubMed; Scopus; Cochrane Central Register of Controlled Trials (CENTRAL).

The research strings were as follows:

- Nerve growth factor;
- NGF;
- Neurotrophins;
- Neuroplasticity;
- Traumatic brain injury;
- NGF AND children;
- NGF AND ophthalmology;
- NGF AND oncology.

Only English-written publications were included. The current investigation mainly concentrated on randomized placebo-control studies, case-control studies, retrospective and prospective observational studies, and systematic reviews and meta-analyses. 

The article selection method was supported independently by three reviewers (LC, SF, and LDS). 

All significant articles discovered were further scrutinized for extra references that did not appear in the preliminary examination.

Review or commentary papers without original data were eliminated, whereas their contents were used to clarify collected information.

All papers not noticeably concerning nerve growth factor were similarly excluded.

The quality of the trials was thoroughly evaluated, and the following potential biases have been assessed: random classification group (selection bias); similarity of patients at baseline concerning the most significant prognostic indicators (homogeneity bias); allocation hiding (selection bias); blinding of workers (performance bias); blinding of outcome valuation (detection bias); partial outcome data (attrition bias); evading of co-interventions (co-intervention bias); report of drop out (drop out bias). 

A total of 142 papers were encountered in the literature search. Among them, after the application of inclusion and exclusion criteria, 101 papers were selected, while 41 papers were excluded.

## 3. Results

### 3.1. Studies on NGF in Central Nervous System’s Diseases

Over the last few decades, neurotrophins collectively have gained major importance in the medical research field since their roles seem to be associated with regulatory functions on the survival, neuroprotection, connection, growth, and differentiation of nerve cells in the peripheral and central nervous systems [24].

Many studies, in vitro and in vivo, demonstrated the central importance of NGF and its ubiquitous production in the nervous system.

Because of its localization and role, the factor itself was subsequently applied for therapeutic purposes in several areas, first of all in the central nervous system. This aspect represents an extraordinary opportunity in the pediatric sphere, where the administration of NGF through different delivery routes demonstrates surprising biological and clinical outcomes. 

#### 3.1.1. Nerve Growth Factor in Traumatic Brain Injury

Severe traumatic brain injury (TBI) is the most common cause of death and acquired disability among children and young adults in industrialized countries. Secondary effects due to brain injury involve different mechanisms such as inflammatory pathways with the production of cytokines, oxygen radicals, and consequent balance between the production and activity of neurotrophins, leading commonly to cell loss and impairment of cholinergic functions. 

In recent years, new pharmacological and non-pharmacological techniques have been developed for the treatment and prevention of secondary damage after severe brain injury.

As demonstrated in a prospective observational clinical study, levels of IL-6 and NGF measured in the cerebrospinal fluid (CSF) of children with severe TBI were higher than those of controls, proving that inflammatory mediators were implicated in the reactive process to TBI. 

Others supported the pivotal role of NGF and doublecortin (DCX) in neurogenesis and neuronal repair; measuring NGF and DCX values in CSF of children with TBI at T1 and T2 (2 h and 48 h later TBI respectively) has shown that children with best outcomes had higher levels of NGF than those with poor outcomes, and the expression of DCX increased only in patients with NGF up-regulation from time T1 to time T2 [25]. Elevated levels of both NGF and DCX were also found in the CSF of a patient with TBI after treatment with NGF. Moreover, NGF seems to correlate significantly with the severity of head injury and better neurologic outcomes [26].

Surprising results came from an experimental study on rats that involved giving intranasal NGF in a TBI model; improvements in motor dysfunction and decreasing neuroinflammation have been observed [27]. 

Intranasal treatment with NGF has also been tested on children, obtaining remarkable outcomes as well since, to date, there are no therapeutic strategies in children affected by severe traumatic brain injury, and the only applicable therapy is palliative. Encouraging changes in Positron Emission Tomography/Computed Tomography (PET/CT), SPECT/CT, Magnetic Resonance Imaging (MRI), EEG, and Visual Evoked Potential (VEP), and improvements in clinical conditions have been reported after intranasal NGF treatment in a 4 years-old child with TBI. 

#### 3.1.2. Nerve Growth Factor in Hypoxic–Ischemic Brain Injury

Hypoxic–ischemic brain injury (HIBI) is a rare but devastating condition characterized by neural loss that often leads to a poor neurological outcome. In children, no innovative therapeutic strategy has emerged recently. In experimental and clinical studies, it has been demonstrated that brain damage might cause an increased expression of neurotrophins in the nervous system, and NGF up-expression or exogenous administration has been associated with the reduction of neural degeneration, peripheral nerve regeneration, and neurological improvements. 

Chiaretti et al. reported positive results in toddlers treated with intraventricular NGF [28]. In particular, after administration via the external catheter into the brain in patients who suffered HIBI with a Glasgow Coma Scale (GCS) of 4 and 5, respectively, the infants showed clinical amelioration in terms of communicative and motor functions, with a change of 4 points in CGS score for each. An electroencephalogram (EEG) repeated after treatment revealed a reduction of slow-wave activity and an increase in higher frequencies. Cerebral perfusion at single photon emission computed tomography (SPECT) was significantly better in specific areas [28,29]. Also, magnetic resonance imaging (MRI) showed a reduction in malacic areas and diffuse cystic cavitation.

NGF, therefore, seems to be a promising factor for approaching children affected by HIBI since its administration is correlated to neuroprotection, neural stimulation, and clinical and radiological improvements without side effects. 

#### 3.1.3. Nerve Growth Factor in Central Nervous System Infections

Meningoencephalitis (ME) is an infection of the central nervous system involving meninges and brain tissue. As all we know, neurotrophins regulate the physiological homeostasis of neuronal cells, stimulating growth and differentiation and favoring neuroprotection. 

In infectious states, changes in neurotrophic factor levels in cerebrospinal fluid (CSF) may reflect an endogenous effort to protect neurons against biochemical and molecular damage. 

In a prospective observational clinical study conducted at Catholic University Medical School of Rome, the researchers evaluated neurotrophin levels in plasma and CSF of 13 children affected by viral or bacterial meningoencephalitis compared to 12 children with non-inflammatory obstructive hydrocephalus. In the CSF and plasma, the amount of neurotrophic factors expression appears increased in comparison to controls; NGF and BDNF levels were higher in children with viral and bacterial ME. Stratifying between etiologies, in viral ME, the mean level of NGF in the CSF was significantly greater than in bacterial ME.

The other interesting finding was a statistically significant association between persistent CT scan abnormalities, worse clinical symptoms, and children with higher NGF levels among those with bacterial ME. 

All these results agree with the pediatric literature about NGF and other neurotrophins since their augmented presence in injured and inflamed sites can be related to their neuroprotective role [30].

A subsequent study showed similar results about high levels of NGF and BDNF in children affected by Epstein–Barr virus meningoencephalitis and their correlation to disease severity and increased numbers of lymphocytes in the CSF, underlining again the possible role played by these factors in the molecular inflammatory cascade.

Supported by these hypotheses and evidence, it has been recently described the outcomes of human-recombinant nerve growth factor (hr-NGF) treatment in an infant six months after a group B Streptococcus (GBS) meningitis [31]. After repeated cycles of intranasal pharmacological administration without side effects, improved clinical outcomes such as ameliorations in facial mimicry, attention, motor reactions, oral motility, and feeding capacity emerged. Corresponding improvements in electroencephalogram (EEG) and magnetic resonance imaging (MRI) were reported; positron emission tomography (PET/CT) and single-photon emission CT (SPECT/CT) showed an increased metabolism in specific areas. 

The surprising results obtained by intranasal administration of hr-NGF confirm the therapeutic potential of human neurotrophins through a safe and painless delivery route.

#### 3.1.4. Nerve Growth Factor in Cerebral Malformations

Microgyria and hemimegalencephaly (HME) are two congenital disorders characterized by abnormal development, malformed histology, and altered morphology of the central nervous system, which determine their clinical peculiarities. 

In both cases, there is evidence of a neuronal migration deficit during a critical period of brain development [32,33], whose mechanism is largely unknown in the case of HME [34], while focal hypoxia and ischemia are believed to be at the basis of HME pathology when occurring during the late stages of cortical migration [33].

The study of Antonelli et al. [32] is very helpful in understanding the role of NGF in HME, analyzing the brain tissue of four infants affected by this condition. They revealed an increase in NGF and its mRNA in the cerebral altered tissue of the study population compared to the control group, as well as an increase in the number of TrkA-positive cells in HME tissue in the face of reduced activity of its effector choline acetyltransferase (Chat) [32]. 

To sum up, dysregulation in neurotrophin balance and receptor expression with relative final activity could play a key role in the etiopathogenesis of altered neuronal migration and differentiation.

TrkA upregulation is also observed in rats with experimentally induced focal microgyria to which Chiaretti et al. [33] administered intraparenchymal NGF since previous animal studies have shown the role of this factor in growth and differentiation of neural crest-derived cells. 

TrkA upregulation was already visible 72 h after NGF administration, as were doublecortin (DCX) levels, which constituted a marker of neurogenesis, and this success confirms that NGF could act as a potential neuroprotective factor blocking or slowing the apoptotic pathways.

#### 3.1.5. Nerve Growth Factor in Autism Spectrum Disorders and Attention Deficit Hyperactivity Disorders

Autism spectrum disorders (ASD) constitute a condition of wide and variegated aspects, not all completely understood, as well as attention deficit hyperactivity disorder (ADHD), whose pathogenesis is not totally known despite it constituting the most diagnosed neurobehavioral disorder of childhood. 

According to recent studies, an imbalance in NGF and the other neurotrophins could represent one of the causative mechanisms at the basis of these diseases [17,34,35,36]. This theory is supported by the evidence of data obtained from the analysis of 31 studies examined, which involved a total of 2627 children affected by ASD [34]; peripheral blood levels of NGF are statistically significantly higher compared to the control group as well as BDNF and VEGF levels. Although it is still not clear what the role of neurotrophins in ASD is, this analysis suggests a connection between factors and illness pathogenesis. Excessive brain development and structural changes in the brains of children affected by autism spectrum disorders, moreover, could be backed by the neurotrophic action of NGF.

Similarly, as reported by Guney et al. [17], increased blood levels of NGF in 44 patients affected by ADHD showed a statistically significant difference compared to the control group. The authors suggest that the reasons for this result include the association between NGF expression and aggressive behavior, NGF production and stressing events being considered one of the possible causative factors of ADHD, and the correlation between NGF levels and brain changes reported in children with ADHD.

In conclusion, dysregulation of neurotrophic factors could have a crucial role in the development of neuropsychiatric diseases.

#### 3.1.6. Nerve Growth Factor in Peripheral Nervous System Disorders

The effects of endogenous neurotrophins on the peripheral nervous system and their roles are not completely clear. Many studies on rats in the last decades aimed to demonstrate the different implications of NGF on spinal cord injuries and their consequences, as well as sympathetic sprouting in the dorsal root ganglia and allodynia and possible amelioration of clinical outcomes [37]. 

In accordance with an accepted hypothesis about the intrinsic etiology of sensorimotor deficit in myelomeningocele (MMC), it has been proposed that damage to spinal neural cells in the early stages of fetal development could be a cofactor in the constitution and consolidation of the malformation [38]. 

In this scenario, a further confirmation of the connection between the peripheral nervous system and MMC has been provided by Chiaretti et al. since BDNF, GDNF, and NGF levels in the CSF of newborns affected by myelomeningocele were higher compared to the mean values of the control group [39].

The above-mentioned neurotrophins play a fundamental role in the synaptic plasticity and modeling after a spinal injury, providing neuroprotection and strengthening some neuroregenerative activity secondary to an upregulation in this condition. 

In 2000, research conducted in Australia and published by Experimental Neurology [37] showed some of several functions of neurotrophins in the peripheral nervous system after an injury; authors found that both NGF, NT3, and BDNF antisera considerably reduced the sympathetic germination in a rat spinal nerve injury model, specifically in dorsal root ganglia involved in neuropathic pain, therefore suggesting the hypothesis that these factors were involved in modulating allodynic response; in particular, they support the idea that NGF may play a role in the sprouting of sympathetic axons in dorsal root ganglia after nerve lesions [37].

Many years later, since the spread of an alternative delivery route of NGF has been tested in the central nervous system, Aloe et al. obtained clinical and molecular results in their cohort of rats with spinal injuries, administrating intranasal NGF. 

The increasing amount of NGF and enhanced expression of nerve growth factor receptors in the spinal cord demonstrated the effective passage through the blood–brain barrier and its related clinical outcomes, such as improving the locomotor deficits, suggesting the possible therapeutic approach of spinal cord injury using this via [40].

Moreover, there is a connection between NGF and the pain pathway. The exposition to NGF produces sprouting of nociceptive fibers and hyperalgesia in adults, while inhibitors produce opposite effects [3]. Finally, a further implication of nerve growth factor on the pain route is demonstrated by the impact of Fasinumab, an antibody against NGF, on improvement in pain scores [41].

The main characteristics of the selected studies are resumed in Table 1.

### 3.2. Studies on NGF in Ocular Diseases

Among the numerous clinical applications of NGF, its use as a therapeutic agent in ophthalmology is certainly one of the most explored [42]. The early evidence of its role comes back to 1979 when Turner et al. proved that the retinal cells of goldfish had receptors for NGF [43]. As a matter of fact, NGF receptors are ubiquitously distributed in conjunctiva, cornea, retina pigment epithelium, photoreceptors, and retinal ganglion cells in the eye [44].

NGFmRNA is detectable in the cornea, iris, ciliary body, and lens, and NGF is measurable in the aqueous humor as well [24]. The principal determinant of NGF action is the relationship between TrkA and p75, which are located on cell surfaces concurrently [45]. Several circumstances, such as ocular trauma, inflammation, and allergy, may affect the presence of these receptors in conjunctival cells or corneal cells, ultimately leading to a lack of tissue repair and enhancing impairment in the nervous cells [46,47].

Delivery route options for ocular therapies are topical, spanning from intravitreal, subconjunctival, or sub-tenon until periocular or retrobulbar injections [48]. NGF may be administered topically in a murine form or a recombinant one. The murine NGF was obtained from mice’s submaxillary salivary glands and found applications in several trials [49], whereas Cenegermin is the recombinant form of human NGF (rhNGF) produced in Escherichia coli. Cenegermin became the first drug approved by the FDA to treat neurotrophic keratopathy in adult and pediatric patients over the age of two [50].

Corneal epithelial cells are one of the most densely innervated, and modification of their innervation can eventually cause corneal damage, leading to consequent visual impairment [24]. In 1998, Lambiase et al. demonstrated that exogenous NGF restored corneal integrity in patients with corneal neurotrophic ulcers [51]. In this pilot uncontrolled trial, intraocular applied mouse NGF reinstated corneal integrity in patients with corneal neurotrophic ulcers in a timeframe spanning from 10 to 42 days [51]. In line with these results, Bonini et al. performed a prospective interventional study on neurotrophic keratitis non-responsive to conventional treatments [52]. All their patients had complete healing of the epithelial defects after 12 to 42 days of therapy with mice NGF [52]. In 2007, a further study evaluated the effect of topical mice NGF on 11 patients with corneal neurotrophic ulcers with a specific focus on its side effects and systemic implications [53]. A complete repair of ulcers occurred between 9 and 43 days. No generation of anti-NGF antibodies was observed in a follow-up time of 72 months. None of the patients experienced any side effects except for mild and transient conjunctival hyperemia and photophobia [53]. In recent years, many studies have demonstrated the efficacy and safety of topical rhNGF for treating neurotrophic keratitis. In 2018, the REPARO trial—a phase I/II randomized, double-blinded, multicenter, vehicle-controlled study—evaluated the effectiveness of rhNGF in patients with moderate or severe ulcers [54,55]. The phase I and phase II studies proved that topical intraocular rhNGF (10 or 20 mg/mL), administered 6 drops/day for 56 days, was well tolerated in a sample size of 18 adults first and 156 after [54]. Patients were randomized to rhNGF 10 mg/mL, 20 mg/mL, or vehicle. Most side effects were ocular, mild, and transient and did not require a stop in the treatment process. After 8 weeks of treatment, 43.1% of vehicle-treated patients achieved corneal healing versus 74.5% receiving rhNGF 10 mg/mL and 74.0% receiving rhNGF 20 mg/mL [55]. Afterward, in 2020, Pflugfelder et al. conducted a multicentered, randomized, double-masked, vehicle-controlled trial using 48 patients with neurotrophic keratopathy [56]. They randomized patients 1:1 to Cenegermin 20 μg/mL or vehicle eye drops, six drops daily for 8 weeks of masked treatment. In comparison to the vehicle, rhNGF-treated patients confirmed statistically significant decreases in ulcer size and disease progression rates during masked treatment, documenting a well-tolerated strategy [56]. Recent studies have also supported the pivotal role of Cenegermin in neurotrophic keratopathy in pediatric patients [57]. Due to its non-invasive administration route, rhNGF is preferred to other approaches by parents and physicians in pediatric age [57]. In 2019, Pedrotti et al. described a case of neurotrophic keratopathy occurring after surgery for rhabdomyosarcoma of the jaw in a 3-year-old boy [58]. He underwent radiotherapy and subsequently developed corneal ulcers resistant to conventional therapy. This patient was successfully treated with Cenegermin eye drops six times daily for 8 weeks [58,59]. In 2021, Leto et al. reported a case of a pediatric patient affected by neurotrophic keratopathy with Goldenhar syndrome [59]. This patient presented corneal ulcerations and a small corneal opacity in both eyes. He was treated with Cenegermin eye drops six times daily for 8 weeks. Total healing was accomplished in both eyes after treatment. During the 16-month follow-up period, no epithelial defects, relapses, or concerns were detected [59]. Similarly, Papadopoulos et al. described a case of a 9-year-old patient with corneal ulcers due to a refractory infection [60]. A treatment with rhNGF was applied, and a complete regression of corneal ulcers was achieved within 8 weeks [60]. 

In 2021, Hatcher et al. conducted a retrospective case series study of eight pediatric patients undergoing an eight-week course of Cenegermin therapy [61]. Five patients fulfilled the therapy program, and the same number improved clinically through improved corneal ulcer stage in five cases and visual acuity in two cases [61].

Previous studies have shown that, even when topically applied, NGF reaches significant concentrations in the posterior segment of the eye [62]. This pharmacokinetic propriety could be even more exploited in a pediatric setting where the patient’s compliance may, at times, constitute an issue. Glaucoma is one of the pathologies of the posterior eye segment in which NGF has found application. It is an acquired disease of the optic nerve characterized by specific structural changes with associated visual field defects [63]. More than 60 million people have glaucoma worldwide, making it the second most common cause of blindness [63]. Traditional therapeutic approaches aimed at lowering intraocular pressure. Moreover, recent clinical research aimed at improving the survival of retinal ganglion cells in order to prevent permanent nerve cell loss [63]. In 2009, Lambiase et al. treated three patients with progressive visual field defects despite intraocular pressure control using mice NFG [20]. In this study, the dosage of mice NGF was ten times higher in comparison to the dose used in ocular surface restoration. Patients treated with mice NGF demonstrated long-lasting improvements in the visual field, optic nerve function, contrast sensitivity, and visual acuity. NGF demonstrated neuroprotective effects, inhibiting apoptosis of retinal ganglion cells [20].

Firstly, in 1990, Faktorovich et al. asserted a possible involvement of growth factors in the protection of retinal cells in rodents with retinitis pigmentosa, a genetic condition with progressive photoreceptor degeneration, ultimately leading to the loss of vision [64]. Preclinical trials have confirmed the beneficial impacts of NGF administration on visual function in animals [65]. In fact, in 2005, Lenzi et al. showed that NGF administration had a rescue effect on photoreceptors in a rat model [65]. In 2016, Falsini et al. conducted a trial to explore the potential effectiveness of a short course of NGF eye drop treatment in patients with retinitis pigmentosa [66]. The only side effect reported was a temporary tolerable local corneal irritation. The retinal function, tested at baseline and after treatment, did not show any significant changes in terms of loss. This study supported the safe and effective role of NGF eye-drop administration in patients with this type of disorder [66]. 

This body of scientific evidence has led to the hypothesis that NGF may also play a beneficial role in macular degeneration. Age-related macular degeneration is the most frequent cause of permanent visual loss in the Western world, and its incidence is constantly increasing [67].

In 2009, Lambiase et al. reported a case in which murine NGF was applied to a patient suffering from bilateral macular degeneration [68]. The therapy was applied continually without suspensions for 6 years. Checks were made every 3 months in order to detect any clinical or electrofunctional variations. They reported an improvement both in clinical visual acuity and in the amplitude of the electroretinogram [68].

The reviewed data have demonstrated the safety and effectiveness of NGF in patients with various nervous regenerative indications (Table 2); anyway, clinical experience with this therapy is still recent and limited, especially in pediatric patients.

### 3.3. Studies on NGF in Oncology

Neurotrophins play a critical role in determining tumor cell survival and growth [21,69]. In particular, the NGF receptor (TrkA) is expressed on different specific cancer cells, such as the ones of neuronal origin [69]. Trk receptors are proven to transduce the mitogenic effects of NGF, and their inhibitors are an interesting treatment tool for neurotrophic tyrosine receptor kinase fusion tumors [69]. NGF contributes to cancer inflammation by cytokines release stimulation and can modulate tumor growth by stimulating cancer cell proliferation, promoting apoptosis, producing pro-angiogenic factors, and inducing the epithelial–mesenchymal transition [69]. Thus, NGF signaling pathways may also be useful to predict chemotherapy resistance [21,23,69].

The oncologic role of NGF has been studied since the early 1990s. The first study available in the literature is the analysis of the interaction between the Trk proto-oncogene and the NGF receptor performed by Hempstead et al. [7]. The authors highlighted, for the first time, the importance of NGF receptor-mediated mechanisms in in-vitro cellular differentiation [7].

According to currently available data, an increased signal for activators of the MAP-Kinase pathway, such as NGF, epidermal growth factor (EGF), and platelet-derived growth factor (PDGF), was found in pediatric retinoblastoma cell samples [22]. In addition, some authors proved that, although neurotrophin signaling is generally associated with cell survival, TrkA promotes neuronal death in the developing nervous system [70].

The first interesting in-vitro interventional study was performed by Evangelopoulos et al. [71], who analyzed neurotrophin receptor surface expression of neuroblastoma cells and exposed these cells to recombinant NGF to assess cell line survival and proliferation [71]. They demonstrated different effects of NGF on neuroblastoma cell survival: it decreased proliferation of a p75NTR, but not TrkA-expressing neuroblastoma clone, while an increasing proliferation effect was found in other clones [70,71].

Anyway, most interventional studies were performed by Chiaretti et al. during the last 20 years [72,73,74]. They highlighted the NGF’s role in inducing neoplastic cell differentiation in a human ependymoblastoma cell culture. In fact, their study results suggested that NGF did not promote neoplastic proliferation as observed in non-neuronal origin cells and stimulated ependymoblastoma cells into acquiring the structural characteristics of nerve cells [72].

In addition, NGF administration blocked medulloblastoma proliferation and induced overexpression of p73 in ependymoblastoma clones [72]. Moreover, a combined treatment with NGF plus cisplatin reduced the cytotoxic effect of chemotherapy, confirming the NGF interference with cell proliferation and survival mechanisms [72].

The topical application of NGF was in vivo consistently tested on patients affected by optic glioma [73,74]. Starting from the murine NGF eye drop administration to an adult patient, Chiaretti et al. observed a clinical, electrophysiological, and neuroradiologic repeated improvement in visual function without side effects, and the same data were recorded applying this therapy in two pediatric populations (respectively composed by 5 and 18 patients) [73,74].

All these interesting studies suggest that NGF has a strong potential adjuvant and therapeutic role in pediatric oncology (Table 3), but further studies may be useful in order to know how to personalize and optimize its use according to precision medicine criteria.

### 3.4. Techniques for Biodelivery of Nerve Growth Factor to the Brain

NGF shows significant promise as a therapeutic candidate for neurological disorders. Unfortunately, NGF cannot cross the blood–brain barrier (BBB), making peripheral administration of NGF protein therapeutically impractical. NGF must be delivered in a manner that allows for brain penetration and availability to the basal forebrain cholinergic neurons (BFCNs) to influence BFCN activity and survival. Over the past few decades, various methods have been developed to deliver NGF to brain tissue [75].

In 1998, Jonhagen et al. conducted the first clinical trial aiming to achieve therapeutic NGF levels in the brain by intracerebroventricular (ICV) infusion in three patients [76]. Despite increased nicotinic receptor expression and cognitive improvements, the trial was halted due to induced neuropathic pain and weight loss. They concluded that while long-term ICV NGF administration might have some beneficial effects, the intraventricular route caused adverse side effects that outweighed the benefits [76].

In pediatric cases, NGF has been administered ICV in some compassionate studies to children with severe hypoxic–ischemic trauma without selectively targeting cholinergic function [29,77,78].

Subsequent clinical trials used cells engineered to secrete NGF or adenoviruses carrying the NGF gene. In 2013, Hohsfield et al. demonstrated successful NGF secretion via a lentiviral vector [79]. Concurrently, lentivirus NGF gene delivery to the aged monkey cholinergic basal forebrain for one year showed no systemic NGF leakage, anti-NGF antibody formation, or activation of brain inflammatory markers, pain, or weight loss [80].

Adeno-associated virus (AAV)-based gene delivery has also been used in patients affected by Alzheimer’s disease (AD) to deliver NGF to treat symptoms and progression [81]. In 2018, the same author conducted a phase II clinical trial using AAV vectors expressing human NGF.

In 2005, Tuszynski et al. conducted a clinical trial targeting the BFCNs in eight patients, using autologous fibroblasts engineered to produce NGF. This approach slowed cognitive decline and improved cortical glucose uptake [82].

The in vivo gene therapy showed certain limitations, particularly the permanent genetic modification of brain cells and the inability to control and stop the targeted release.

Recently, clinical trials have begun using encapsulated cell biodelivery (ECB) systems engineered to secrete NGF. The ECB technology aims to combine the therapeutic power of gene therapy with the precision of a safe, retrievable device [83]. In a 2019 study by Mitra et al., EBM cells were placed at the tip of a catheter formed by a semipermeable membrane to allow for NGF and nutrient exchange in the extracellular fluid. Despite encouraging results, the trials slowed due to variability in results from the degeneration of the engineered cells [84].

More recently, 3D printing technologies have been used to construct advanced, precise microstructures for drug delivery [85]. Lipid- and polymer-based delivery systems, magnetic nanoparticles, and quantum dots are also being explored as promising nanotechnological strategies to overcome the current limitations of NGF-based therapies [86,87].

The ocular or intravitreal routes of administration have been extensively discussed in Section 3.2, to which reference should be made.

Intranasal delivery (IND) has overcome the challenge of systemic delivery through both oral and parenteral routes into the brain. Transport to the brain can occur via extracellular and intracellular pathways and through diffusion in the perivascular and perineural spaces of the olfactory and trigeminal nerves. This is a non-invasive method for direct brain drug delivery [87,88]. Relevant models of neurological pathologies where IND-NGF efficacy has been attempted include cerebral ischemia, AD, traumatic brain and spinal cord injuries, epilepsy, amyotrophic lateral sclerosis, hypogonadism related to premature aging, and depression.

Additionally, transcranial direct current stimulation (tDCS) has been reported to be neuroprotective in many neurological diseases, such as motor deficits and cognitive impairment. Curatola et al. studied the effects of combined treatment of intranasal human-recombinant NGF (hr-NGF) and tDCS on brain functions of three children with chronic vegetative state secondary to out-of-hospital cardiac arrest (OHCA) [89].

Furthermore, some authors described the connection between the acupuncture and the neurotrophins, especially NGF. This method is a component of Traditional Chinese Medicine (TCM) used to treat pain, inflammation, motor dysfunction, mood disorders, and seizures based on the stimulation of various types of sensory nerve fibers. More recently, different classes of molecules, including neurotransmitters, cytokines, and growth factors, have also been identified as potential mediators for specific acupuncture outcomes. They underlined the role of NGF, the archetypal member of the neurotrophin family, as a mediator of acupuncture effects in the central nervous system and as a regulator of sensory and autonomic functions [90,91].

The main characteristics of the available studies on the therapeutic use of NGF are resumed in Table 4.

## 4. Conclusions and Future Perspectives

In conclusion, the therapeutic potential of nerve growth factor in addressing pediatric pathologies is promising, yet it remains challenged by delivery obstacles, particularly the blood–brain barrier in the case of the central nervous system. Current research has made significant strides in developing innovative delivery methods such as gene therapy, encapsulated cell biodelivery systems, and intranasal administration. While these approaches have shown varying degrees of efficacy and safety, the risks associated with long-term and invasive treatments require further refinement and the development of further trials as, unfortunately, the application and administration of NGF still remain a very narrow scientific field whose limits also lie in the small number of published studies.

To date, therefore, we can say that the preferred way of administration for reaching the central nervous system in the pediatric setting remains the intranasal route, especially considering the need to use an effective but non-invasive route.

However, it will take several years before reaching new standards, as it is essential to have new, complete, and well-performed clinical studies with an ever-increasing number of patients to fully understand the benefits and potential side effects of NGF therapies in pediatric patients.

Future research should focus on optimizing non-invasive delivery methods and enhancing the specificity and control of NGF targeting. Advances in nanotechnology, 3D printing, and genetic engineering hold substantial promise for overcoming existing limitations.

Ultimately a multidisciplinary approach to integrating these advanced technologies may pave the way for effective and safe NGF-based treatments for pediatric neurological disorders.

## Figures and Tables

**Table 1:** Summary of studies on NGF in pediatric central nervous system diseases.

Disease	Role of NGF	NGF Type and Dosage	Patient’s Age	Delivery Route	Study Outcome	Side Effects	References
Hypoxic–ischemic brain injury	Neuroprotection	A total of 1 mg 2.5S NGF once a day for 10 days	Eight months Thirteen months	Intraventricular (IV)	Amelioration in motor and cognitive functions and state of consciousness, improvement in GCS, SPECT, EEG.	Not reported	[28]
	Neuroprotection	A total of 1 mg 2.5S NGF once a day for 10 days	Eight months Thirteen months	IV	Amelioration in motor and cognitive functions. Recuperation of vocalization improvement in perfusion in SPECT, reduction in slow wave activity, and increase in higher frequencies in EEG	None	[29]
Traumatic brain injury	Neuroprotection	None	Twelve children from 3 to 13.6 years old	None	Upregulation of NGF after TBI is associated with good outcomes. Higher levels of NGF at T1 e T2 in children with best outcomes. Increase in DCX in patients with NGF upregulation.	Not applicable	[25]
	Neuroprotection	None	Twenty-seven children from 1.3 to 15.6 yo	None	Early NGF and IL-1β concentrations (T1) correlated significantly with the severity of TBI. Higher NGF and IL-6 and lower IL-1β expression at T2 were associated with better neurologic outcomes.	Not applicable	[26]
	Neuroprotection	A total of 50 mcg/kg hr-NGF	Twenty postnatal day rats	Intranasal	Acute intranasal NGF prevented the onset of TBI-induced motor disabilities and decreased reactive astrogliosis, microglial activation, and IL-1β content.	Not reported	[27]
Hemimegalencephaly		None	Four children from 4 to 32 months	None	Significative difference in NGF levels between HME and control tissues	Not applicable	[32]
Meningo- encephalitis		A total of 0.1 mg/kg of hr-NGF 3 times daily for 7 days for 5 cycles	Seven-week-old infant	Intranasal	Improvements in PET/CT, SPECT/CT, MRI, and EEG. Improvements in facial mimicry, attention, motor reactions, oral motility, and feeding capacity.	None	[31]
Myelomeningocele	Stimulation of sprouting, synaptic plasticity, and reorganization	None	Fourteen newborns	None	Analysis of NGF expression in CSF of patients before neurosurgical operation: increase in NGF compared to the mean level of the control group	Not applicable	[39]
Focal microgyria	Neural repair	2.5S NGF, purified and lyophilized	Fifteen newborn Wistar rats with induced polymicrogyric lesions on the frontal cortexes	None	A significant increase in TrkA level was found in microgyria + NGF 7 days group compared to the others. Increased TrkA level was already visible in microgyria 72 h after NGF administration. P75 level is decreased after NGF administration. DCX levels increased 7 days after NGF administration.	Not applicable	[33]
Spinal nerve injury	Neuroprotection	A total of 10 μL of purified NGF in healthy rats (single administration) and daily treatment for 3 weeks in a model of spinal cord injury	Forty-two male Sprague-Dawley rats with intact and injured spinal cord	Intranasal	Increased content of NGF and enhanced expression of NGF receptor in the spinal cord 24 h after a single IN administration of NGF in healthy rats improved deficits in locomotor behavior and increased spinal content of both NGF and NGF receptors in a model of spinal cord injury.	Not reported	[40]
	Sprouting in the dorsal root ganglia	Antisera specific to neurotrophins intraperitoneally twice a week for 2 weeks, 10 mL/kg at first injection, and 5 mL/kg at subsequent injections	Male Sprague–Dawley rats	Not applicable	Antisera significantly reduced the sympathetic sprouting and the formation of baskets. L5 spinal nerve lesion induced a significant increase in foot withdrawal responses to von Frey hair stimuli, which was attenuated by treatment of antisera to neurotrophins with a different time sequential	Not applicable	[37]
ADHD	Regulator of neuronal function	none	Forty-four children	Not applicable	The mean serum NGF levels of the ADHD patients were significantly higher than those of the controls.	Not applicable	[17]
Autism	Possible role in onset and/or development of autism spectrum disorders	none	A total of 2627 children with autism spectrum disorders	Not applicable	Blood levels of BDNF, NGF, VEGF in children with autism were significantly higher than those of healthy controls.	Not applicable	[34]

**Table 2:** Summary of studies on NGF in ocular diseases.

Disease	Role of NGF	NGF Type and Dosage	Patient’s Age	Delivery Route	Study Outcome	Side Effects	References
Glaucoma	Restore retinal and optic nerve function	Mice NGF. Four daily applications of a 200 μg/mL solution for 3 months.	Three patients. Mean age 69 years.	Topical intraoular administration	Better functionality of the inside retinal layer and improvement in the parameters of the post-retinal neural conduction and visual acuity.	A transient burning sensation in 1 patient	[20]
Corneal Ulcers	Reestablish corneal integrity	Mice NGF. Daily applications of a 200 μg/mL solution for 6 weeks.	Twelve patients. Mean age 31 years	Topical intraocular administration	Improved corneal sensitivity. Visual acuity increased progressively during treatment.	None	[51]
	Reestablish corneal integrity	Mice NGF. Daily applications of a 200 μg/mL solution until ulcer healing.	Forty-three patients. Mean age 44 years	Topical intraocular administration	Resolution of the epithelial defect with a treatment. Improved corneal sensitivity and visual acuity as well.	Hyperemia and periocular pain in the first days of treatment.	[52,53]
	Restore corneal integrity	Mice NGF. Several daily applications of a 200 μg/mL solution until ulcer healing.	Eleven patients. Mean age 49 years	Topical intraocular administration	A complete repairing of ulcers occurred	Mild and transient conjunctival hyperemia and photophobia	[53]
	Restore corneal integrity	rhNGF (10 or 20 mg/mL), administered 6 drops daily for 56 days	Eighteen patients. Mean age 59 years	Topical intraocular administration	Corneal resolution in patients with moderate to severe corneal ulcers.	Adverse effects were mild, time-limited, and eye-limited.	[54]
	Restore corneal integrity	rhNGF (10 or 20 mg/mL), administered 6 drops daily for 56 days	A total of 156 patients Mean age 60 years	Topical intraocular administration	After 8 weeks of treatment, 43.1% of vehicle-treated patients achieved corneal healing versus 74.5% receiving rhNGF 10 mg/mL and 74.0% receiving rhNGF 20 mg/mL.	Adverse effects were mild, time-limited, and eye-limited.	[55]
	Restore corneal integrity	rhNGF 20 mg/mL administered 6 drops daily was compared with vehicle eye drops for 8 weeks	48 patients Mean age 65 years	Topical intraocular administration	Corneal resolution was higher among patients treated with rhNGF compared with the vehicle.	Mostly local, mild, and transient.	[56]
	Restore corneal integrity	rhNGF eye drops 6 times daily for 8 weeks	A 3-year-old boy	Topical intraocular administration	Corneal resolution	None	[58]
	Restore corneal integrity	rhNGF eye drops 6 times daily for 8 weeks	One infant with Goldenhar syndrome	Topical intraocular administration	Corneal resolution	None	[59]
	Restore corneal integrity	rhNGF eye drops 6 times daily for 8 weeks	A 9-year-old patient	Topical intraocular administration	Complete resolution of the corneal surface and an improvement in visual acuity.	None	[60]
	Restore corneal integrity	rhNGF eye drops 6 times for 8 weeks	Eight pediatric patients. Mean age 8 years.	Topical intraocular administration	Improvements in corneal ulcer stage and visual activity.	Ocular pain, difficulty sleeping, and continued corneal thinning	[61]
Retinitis Pigmentosa	The potential effectiveness of a short course NGF eye drops treatment	Mice NGF. 10 days daily administration as eye-drops for a total dose of 1 mg NGF/patient	Eight patients. Mean age 49 years	Topical intraocular administration	Short-course administration of NGF eye drops produced neither significant adverse effects nor visual function losses	A temporary tolerable local corneal irritation.	[66]
Macular degeneration	Beneficial effect of NGF in arresting macular degeneration	Mice NGF. Three times daily applications of 200 μg/mL solution for 2 separate periods of 1 year and 5 years in the right eye	A 94-year-old female	Topical intraocular administration	Improvement in visual acuity and in the amplitude of the electroretinogram	None	[68]

**Table 3:** Summary of studies on NGF in oncology.

Disease	Role of NGF	NGF Type and Dosage	Patient’s Age	Delivery Route	Study Outcome	Side Effects	References
Neuroblastoma	Arrest of neoplastic cell proliferation (murine cells)	Recombinant NGF	-	In vitro incubation	Diminishing effect on the proliferation of p75NTR+/TrkA- neuroblastoma cells	None	[71]
Ependymoblastoma	Arrest of neoplastic cell proliferation	Murine NGF, 1 ng/mL to 100 ng/mL for 48 h	14	In vitro incubation	Reduction in ependymoblastoma cell survival	None	[72]
Medulloblastoma	Arrest of neoplastic cell proliferation	Murine NGF, 1 ng/mL to 100 ng/mL for 96 h	4	In vitro incubation	Reduction in medulloblastoma cell survival	None	[72]
Optic Gliomas	Visual function protection	Murine NGF, 1 mg three times/day for 10 days	4.6–18.5 years	Topical (eye)	Improvement in visual function	None	[73]
	Visual function protection	Murine NGF, 0.05 mg three times/day for 10 days	2 to 23 years	Topical (eye)	Improvement in electrophysiological and visual subjective measures	None	[74]

**Table 4:** Summary of therapeutic use of NGF and Techniques for biodelivery to the brain.

Delivery Route	Type of Study	Patient’s Age	Disease	Study Outcome and Results	Side Effects	References
Intranasal	Review	Not applicable	CNS disease	Study of experimental factors, such as head position, volume, method of administration, and formulation parameters, that can influence formulation deposition within the nasal passages and pathways followed into the CNS.	None	[87]
	Review	Not applicable	CNS disease	Perineural and perivascular spaces of the olfactory and trigeminal nerves are involved in brain delivery, and cerebral perivascular spaces are involved in widespread brain distribution.	None	[88]
	Review	Not applicable	Down syndrome, Alzheimer disease	The therapeutic potential of hNGFp for the treatment of dementia that is progressively associated with Down syndrome.	None	[92]
	Case series	Three children	Chronic vegetative state secondary to out-of-hospital cardiac arrest	The combined treatment with hr-NGF and tDCS improved functional (PET and SPECT) and electrophysiological (EEG and PSD) assessment.	None	[89]
	Case series	Three children aged 3–10 years old	Unresponsive wakefulness syndrome (UWS) secondary to severe traumatic brain injury (TBI)	Administration of intranasal hr-NGF improved functional (PET and SPECT), electrophysiological (EEG and PSD), and clinical assessment.	None	[93]
Intraventricular	Case report	Three patients	Alzheimer’s disease	Evaluation of cognitive amelioration after treatment with intracerebroventricular NGF.	Two negative side effects: constant back pain was observed in all 3 patients and a marked weight reduction in the first 2 patients	[76]
	Case series	Two infants aged 8 and 13 months	Hypoxic–ischemic brain damage secondary to prolonged cardiorespiratory arrest	The effects of intraventricular NGF and its role in both cerebral perfusion and neurogenesis. NGF treatment resulted in an improvement in regional cerebral perfusion.	None	[78]
	Case series	Two infants aged 9 and 8 months	Hypoxic–ischemic brain damage secondary to prolonged cardiorespiratory arrest	The effects of intraventricular NGF and its role in both cerebral perfusion and neurogenesis. NGF treatment resulted in an improvement in regional cerebral perfusion.	None	[77]
Cell engineered	Review	Not applicable	Neurodegenerative disease	Compared different transfection and transduction methods to generate NGF-secreting primary rat monocytes. They demonstrate that lentiviral infection and Bioporter can successfully transduce/load primary rat monocytes and produce effective NGF secretion.	None	[79]
	Review	Not applicable	Alzheimer’s disease	Prevention of neuronal loss with early life BDNF treatment in mutant mice expressing two amyloid precursor protein (APP) mutations associated with early-onset familial Alzheimer’s disease.	None	[80]
	Phase 1 Clinical Trial	Three patients	Alzheimer’s disease	Evaluation of safety, tolerability, and initial efficacy of three ascending doses of the genetically engineered gene-therapy vector adeno-associated virus serotype 2 delivering NGF. Brain autopsy tissue confirmed long-term, targeted, gene-mediated NGF expression and bioactivity.	None	[81]
	Phase 1 Clinical Trial	Eight patients	Alzheimer’s disease	Phase 1 trial of ex vivo NGF gene delivery in eight individuals with mild Alzheimer’s disease, implanting autologous fibroblasts genetically modified to express human NGF into the forebrain. Cognitive assessment suggested improvement in the rate of cognitive decline. Serial PET scans showed increases in cortical 18-fluorodeoxyglucose after treatment. Brain autopsy of one subject suggested robust growth responses to NGF.	None	[82]
	Review	Not applicable	Parkinson’s disease	Transplantation of encapsulated, GDNF-secreting cells as a strategy for ex vivo cell-based gene deliver	Not applicable	[83]
	Review	Not applicable	Alzheimer’s disease	To describe available experimental and clinical data related to AD therapy, priming to gain additional facts associated with the importance of NGF for AD treatment, and encapsulated cell biodelivery (ECB) as an efficient tool for NGF delivery.	Not applicable	[84]
3D printing technologies	Review	Not applicable	Not applicable	To propose the Biocage, a customizable implantable local drug delivery platform was used; it was fabricated using the Nanoscribe Photonic Professional GT 3D laser lithography system, a two-photon polymerization (2PP) 3D printer	Not applicable	[85]
Nanotechnology	Review	Not applicable	Alzheimer’s disease	To describe main therapeutic approaches that have been developed for NGF delivery targeting the brain, from polymeric implants to gene and cell-based therapies, focusing on the role of nanoparticulate systems for the sustained release of NGF in the brain as a neuroprotective and disease-modifying approach toward AD.	Not applicable	[86]

## Data Availability

Not applicable.
